# The Medical Profession

**Published:** 1854-04

**Authors:** 


### THE MEDICAL PROFESSION.

If we credited all that the croakers say, we should conclude that our profession, at home and abroad, is in a very bad way. The English medical journals are full of complaints—Quackery is described as being everywhere prosperous in that realm; all the desirable places are filled with bad men, and all the good men are out of place; pretenders are growing rich, while the physicians of real merit are starving. And these grumbletonians point to America as the "paradise of legitimate medicine!" Their brethren on this side of the "great waters" must be amused. Here, where the profession of medicine is becoming every day more and more degraded; where the quacks have it all their own way; where "old fogyism" is triumphant, and "young physic" neglected, slighted, ignored—this the paradise of doctors! Truly, if these things are so, there can hardly be a worse calling than our time-honored profession. But they are not true. Things might be better than they are, but they are not half so bad as these hypochondriacs represent them to be. Quackery is prevalent and pestilent, but it is not over-riding and over-shadowing legitimate medicine. Quacks are not getting all the good fees, though they are receiving too many. Medical science was never before making so sure and rapid progress, and it is establishing itself more and more securely in the confidence of men. The medical profession never held a higher rank among human avocations than it does at the present day, and was never entitled to a higher one. We do ourselves injustice by these exaggerated accounts of the success of quackery, and these complaints about the decline of the profession. "How comes it to pass," we know it was asked a long time ago, "that no man is contented with his lot?" Perhaps it is not well that we should be contented with our present state, for then we should not improve; but on the other hand we gain nothing by these sorrowful pictures which we draw of the condition of legitimate medicine, and which must be infinitely pleasing to the "irregulars."

